# Severity and death

**DOI:** 10.1007/s11019-024-10193-z

**Published:** 2024-02-08

**Authors:** Adam Ehlert

**Affiliations:** Department of Public Health and Caring Sciences, Centre for Research Ethics and Bioethics (CRB), Uppsala University, Uppsala, Sweden

**Keywords:** Severity, Priority setting, Health care, Death, Absolute shortfall

## Abstract

This article discusses the relationship between two theories about the badness of death, the Life-Comparative Account and the Gradualist Account, and two methods of operationalizing severity in health care priority setting, Absolute Shortfall and Proportional Shortfall. The aim is that theories about the badness of death can influence and inform the idea of the basis of severity as a priority setting criterion. I argue that there are strong similarities between the Life-Comparative Account and Absolute Shortfall, and since the Life-Comparative Account is one of the most reasonable accounts of the badness of death, this provides some support for using Absolute Shortfall. I also argue that it is difficult to find support for Proportional Shortfall from theories about the badness of death, and also, that it is difficult to find support for Gradualist Account from theories about severity.

## Introduction

In any healthcare system, priority setting is unavoidable.^1^ Many publicly run healthcare systems have formulated explicit priority setting criteria to determine how scarce resources should be distributed and which patient groups should be prioritized. One such criterion is prioritizing based on the severity of illness,^2^ which means that the more severe a condition is, the higher priority it should receive. However, the notion of severity is under-theorized, with an unclear normative notion (Barra et al. 2020).

One aspect that needs further inquiry is how severity is related to death (Barra et al. 2020). Intuitively, death or risk thereof is at least *part* of what makes a condition severe. There is a wide philosophical literature on death and its (proposed) badness (Bradley et al. 2013; Kagan 2012; Nagel 1970). Several authors have argued that these ideas have relevance for priority setting (Gamlund and Solberg 2014, 2019;Norheim 2019; Solberg and Gamlund 2016), because they can give theoretical support to practice, tell us something about which values are central, and contribute to discussions about how future health benefits should be weighted (Gamlund and Solberg 2014). However, a lot of such work remains to be done.

To begin to understand the relationship between severity and premature death, we can look at operationalizations of severity in healthcare priority setting. There are currently two main ways of describing the severity of different conditions. Call these *Absolute Shortfall* and *Proportional Shortfall*.^3^ They are both based on the Quality-Adjusted Life Years (QALY) measurement (Broome 1993) and express the amount of good life years (QALYs) that are lost due to the condition. 1 QALY represents one life year at perfect health. A detailed explanation of Absolute and Proportional Shortfall will follow in “Absolute shortfall”.

Essentially, Proportional Shortfall is less sensitive than Absolute Shortfall to the size of the loss, that is, the amount of QALYs lost to a condition. Absolute Shortfall will generally imply that conditions that result in a patient group dying young are worse than what Proportional Shortfall will imply. Is this an advantage or disadvantage with Absolute Shortfall?

Many people have the intuition that it is, all else being equal, worse to die at 40 than at 80. Intuitively, there is at least *something* worse with this death. In trying to answer what (if anything) is bad about a certain death, we can turn to the aforementioned philosophical debate about *the badness of death*. Several different theories about the badness of death provide us with different explanations as to why death is bad (for the one who dies), and also when (and why) it is worse to die. These theories can generally be separated into two broad categories: Epicureanism and Deprivationism (Epicurus 1940; Nagel 1970). Epicureanism holds that death is never bad (or good) for a person, while Deprivationism holds that death *can be* bad (or good) for a person and that this is so because it can deprive her of future well-being. It should be noted that while the view has taken inspiration, as well as received its name, from Epicurus, it is doubtful whether Epicurus himself would be considered a contemporary Epicurean. It is, in some ways, unfortunate that the contemporary philosophical view shares his name. In this article, “Epicureanism” refers to the contemporary philosophical view. Deprivationism can further be separated into Life-Comparative accounts and Gradualist accounts. Life-Comparative accounts hold that death *can be* bad for a person and that this is so because it can deprive her of future well-being, and further, that the amount of badness can be derived from the amount of future well-being she is deprived of (Broome 2004; Feldman 1992; Nagel 1970). Gradualist accounts are similar to Life-Comparative accounts, but where Life-Comparative accounts hold that the badness of death typically decreases over the course of a life, Gradualist accounts hold that death is bad to different degrees at different times (Belshaw 2005; DeGrazia 2003, 2007; McMahan 2002). More specifically, they hold that the badness of death does not have to be strictly decreasing over the course of a life—it can be modified by some other factor. Presently, it will suffice to say that Life-Comparative and Gradualist accounts will give the same verdict in some cases, but not in other cases. There is at least *some* agreement between Life-Comparative and Gradualist accounts. Most importantly, they both hold that death can be bad. Epicureanism denies that death can be bad, and there is hence no agreement between Epicureanism and Deprivationism (be it Life-Comparative or Gradualist accounts). Also, it may seem that Epicureanism goes against the ethos of health care, since one of the interests of health care is prolonging the lives of people, or, more loosely speaking, “avoiding death”. For that reason, I will leave Epicurean accounts out of this discussion.^4^,^5^

This paper aims to abridge the discussion of severity with the discussion of the badness of death. This aim, framed as a question, is: Does the fact that a disease leads to premature death add to severity? To find out, we need an account of the badness of death that is aligned with the most plausible ideas of priority setting based on severity. I will examine different accounts of the badness of death, and investigate what kind of notion of severity they might support (and vice versa). In the next section, I will go over some preliminaries. I will then describe Absolute and Proportional Shortfall. I will then describe two Deprivationist theories, the Life-Comparative Account (LCA) and the Time-Relative Interest Account (TRIA). I will then examine how and in what way these different theories can give support to each other. I will argue that LCA gives support to Absolute Shortfall (and vice versa) and that no current account of the badness of death can give support to Proportional Shortfall. I will further argue that no account of severity can be coupled with TRIA and that this gives us reason to believe that TRIA is difficult to use in health care, at least presently. My conclusion is that if we accept a deprivationist theory, this provides us with a reason to subscribe to the idea of Absolute Shortfall as an operationalization of severity. In practice, this implies that we ought to judge a health condition more severe than another if that condition contributes to a bigger absolute shortfall of QALYs. This allows us to give higher priority on the basis of severity to conditions that lead to premature death.

## Preliminaries

As mentioned above. I will use the QALY scale of ranking conditions on a scale from 0 to 1, where 1 represents perfect health, and 0 represents death (or a condition that is equally bad as death, but not worse). 1 QALY can be equal to 1 year at QALY level 1, 2 years at QALY level 0.5, 4 years at QALY level 0.25, etc. In the cases where it is of relevance how many actual life years are involved in a case, this will be made explicit. It will be stipulated that the total QALYs in a reference life is 80.

The currency that is measured on this 0–1 scale is what I will refer to as *health-related quality of life*. This is shorthand for those parts of a person’s well-being that can be affected by illness. We assume that the well-being of a life consists of several different things and that health is one of these things. As an example, let’s say that I win the lottery tomorrow. We can assume that this would add some units of well-being to my life, i.e. make my life better, but at the same time, we assume that the well-being added from my winning the lottery is not health-related. Similarly, if I get cancer tomorrow, we can assume that this subtracts some units of well-being from my life, i.e. makes my life worse, and at least some of this well-being is health-related. If I have cancer and get miraculously cured of it tomorrow, this makes my life better in several ways, and some of them are health-related. This is, of course, a complex issue, and whether health is at all separable from general well-being is subject to substantial debate (Bognar 2008; Brock 2002; Broome et al. 2002). For our purposes, I will simply assume that a measurement of health-related quality of life makes some sense, and that changes in health state in some way correlate with changes in well-being.

A *condition* is simply a disease, disability, or injury that negatively affects someone’s health-related quality of life, primarily by a decrease in level of functioning and/or loss of life via death.

When speaking of the *prognosis* of a condition, this refers to the total number of QALYs that obtains as a result of a condition. If the prognosis of a condition is 10 QALYs, that can mean that a patient with the condition will live for 10 years at full health and then die. It can also mean that a patient with the condition will live for 20 years at health level of 0.5 and then die. A has a better prognosis than B, or condition *x* has a *better* prognosis than condition *y*, if A rather than B or *x* rather than *y* involves more QALYs.

When speaking about death, I will assume what has been called *the termination thesis* (Feldman 2000), that is, that death is the permanent end of our existence. I will simply leave aside the debate about whether we are in some way immortal, or continue to exist after our death. I will assume that, following death, a person no longer exists, and therefore has no more well-being.^6^

It is important to note that both Life-Comparative and Gradualist theories are compatible with the idea that death can be *good*. Remember, what Epicurean theories say is simply that death is *neither good nor bad*. If we are not Epicureans, we probably think that death is in most cases bad. But, if one’s life is really bad (maybe it has a net well-being level of less than 0), for instance, if one lives with a very painful chronic condition, it is not absurd to state that death could be *good*.

## Absolute shortfall^7^

Absolute Shortfall measures the amount of QALYs one is expected to lose because of a certain condition. Simply put, the more QALYs that are lost, the more severe the condition is judged. In calculating absolute shortfall, we look at how many QALYs are expected to remain at the time a condition occurs. (In our examples, we stipulate that the expected total QALYs for all patient groups be 80, but again, this is simply to make the examples easier to follow.) Absolute Shortfall is connected to the prognosis of a condition in the way that a better prognosis will, all else being equal, result in fewer QALYs lost than a worse prognosis. The following example illustrates how absolute shortfall works when evaluating the severity of deadly diseases.

*The Absolute Shortfall of deadly disease*. A condition affects 40-year-olds and has a prognosis of 5 QALYs. This patient group will then lose (80-40-5) = 35 QALYs. Another condition affects 70-year-olds, also with a prognosis of 5 QALYs. This group will lose (80-70-5) = 5 QALYs. Since the 40-year-olds will lose more QALYS this condition is judged more severe than the condition affecting 70- year-olds, even if the prognosis is the same.

For deadly diseases with equal prognosis, conditions that affect younger groups will be judged more severe than conditions that affect older groups. How big the effect of this is will depend on if the prognosis is dependent on age. It is reasonable to think that in some situations older people have a worse prognosis than younger. This will give older people a slightly bigger absolute shortfall than if the prognosis would be the same.

*The Absolute Shortfall of chronic, non-deadly disease.* A group of patients averaging 40 years old is affected by a condition that will result in a loss of functioning and reduced health-related quality of life from 1 to 0.8. The absolute shortfall is equal to remaining lifetime times the loss in health-related quality of life (1-0,8=0,2). This will result in an absolute shortfall of 40*0,2 = 8 QALYs. Another group, averaging 70 years of age, is affected by another chronic condition, that also reduces health-related quality of life from 1 to 0.8 For this group, the absolute shortfall is 10*0,2 = 2 QALYs.

Similarly, for chronic conditions, the absolute shortfall is higher for conditions that occur early in life.

*The Absolute Shortfall of passing disease.* Two groups of patients, one averaging 20 years old and the other averaging 70 years old, are both affected by a condition that results in a temporary loss of function that reduces health-related quality of life by 0.5 for 4 years. For both groups, this will result in an absolute shortfall of 2 QALYs.

Absolute Shortfall will give higher priority to those that lose larger parts of their future life because of disease. Because of this, even if age is not a built-in criterion, Absolute Shortfall will often imply that diseases that affect younger people will be seen as more severe than diseases that affect older people.

## Proportional shortfall

Proportional Shortfall, as the name implies, measures the proportion of remaining QALYs that one can be suspected to lose because of a condition. Proportional shortfall holds that it is equally severe to lose 20 of 40 remaining QALYs as 2 of 4 remaining QALYs. This understanding of severity is also linked to loss of future good life but in relative, and not absolute, terms. The following example illustrates how proportional shortfall evaluates the severity of deadly diseases.

*The Proportional Shortfall of deadly disease.* A condition that affects a group of 40- year-olds and has a prognosis of 10 QALYs will give a proportional shortfall of (80-40-10)/(80-40) = 30/40 = 75%. A condition that affects a group of 70-year-olds and has a prognosis of 2,5 QALYs will also give a proportional shortfall of (80-70-2,5)/(80-70) = 7,5/10 = 75%. Put differently: it is equally severe that a 40-year-old dies at 50 as that a 70-year-old dies at 72,5.

As we can see, Proportional Shortfall is less sensitive to the absolute size of the loss, that is, the amount of QALYs lost. We instead look at the percentage of future life that is lost. Similar to the absolute shortfall, the proportional shortfall is also often bigger for younger patient groups. Proportional Shortfall tries to temper the differences so that the proportional difference is smaller than the absolute difference.

*Another deadly disease*. A condition affects 40-year-olds and has a prognosis of 5 QALYs. This group will lose (80-40-5) = 35 QALYs. The proportional shortfall is 35/40 = 87,5%. Another condition affects 60-year-olds and also has a prognosis of 5 QALYs. This group will lose (80-60-5) = 15 QALYs. The proportional shortfall is 15/20 = 75%.

The idea with Proportional Shortfall is to try to remove *some* of the effects of age on severity. In the above example, the relationship between the absolute shortfall is 2.3 (35/15), but the relationship between the proportional shortfalls is 1.16 (87.5/75).

*The Proportional Shortfall of chronic, non-deadly disease.* A group of patients averaging 40 years old is affected by a condition that results in a loss of functioning and reduced health-related quality of life from 1 to 0.8. The proportional shortfall is equal to the loss in health-related quality of life, that is, 0,2 (20%). Another group, averaging 60 years of age, is affected by another chronic condition, that also reduces health-related quality of life from 1 to 0.8. For this group, the proportional shortfall is also 0,2 (20%).

The proportional shortfall for chronic conditions is independent of age. Chronic conditions affecting younger people will not be seen as more severe than chronic conditions affecting older people. Using Proportional Shortfall, how long one lives with a chronic condition will not affect our evaluation of the severity of the condition and therefore make no difference in priority setting.

*The Proportional Shortfall of passing disease.* Two groups of patients, one averaging 20 years old and the other averaging 70 years old, are both affected by a condition that results in a temporary loss of function that reduces health-related quality of life by 0.5 for 4 years (2 QALYs). The proportional shortfall is bigger for the older group (2/10 = 20%) than the younger group (2/60 = 3,3%).

Proportional Shortfall deems a loss of a given amount of QALYs more severe the older the patient is. This is because the QALYs lost will constitute a bigger proportion of the expected remaining QALYs the older the patient is. Using Proportional Shortfall, passing conditions that affect older people will therefore be seen as more severe than passing conditions affecting younger people.^8^

## The badness of death: the life-comparative account

Influentially, Nagel (1970) has argued that death is bad because it deprives us of something, namely our future life. This is the reason the theory has been called Deprivationism. A popular version of Deprivationism is the *Life-Comparative Account* (LCA), which compares the life where I die with the life where I do not die.^9^ If I die at 40, we compare that life with the life I would have had if I did not die at 40. Let’s say that if I did not die at 40, I would live until 80. Then, death *deprives* me of 40 years of life.

More specifically, it could be said that death deprives me of my future *well-being*. When I am dead, I will have no well-being level. I will not exist.^10^ So, let’s say that I am 40, and have had 100 units of well-being so far. We can suspect that if I live to become 80, I will have had an additional 100 units of well-being. But if I die at 40, I will not get to have these 100 units. Death deprives me of 100 units of well-being. That is why death is bad.

On LCA, the badness of death can be formulated simply:

LCA: The badness of death *b* at *t* is a function *b*=*(f)w*, where *w* is the amount of well-being lost from death.

LCA is an account of death's badness, but more specifically, it is an account of why death is bad for *the one who dies*. Why is death bad for *me*? Even if LCA was false, it could still be the case that my death would be bad for *others*.^11^ Even the Epicurean, who denies that death can be good or bad, accepts this. LCA also gives no verdict about my *dying*. Even the Epicurean can agree that the circumstances surrounding my death can be painful for me.^12^ Nor does LCA say anything about which *attitude* I should take toward death. It does not say that I should (or shouldn’t) fear death, or that the mere fact that I am a mortal being (and will die) is bad for me.^13^ LCA regards not others’ attitudes towards my death, not the circumstances surrounding my death, not my attitude to my death, but the *event* of my death.

As expressed by Nagel, LCA is quite simple. Death is bad for me because it deprives me of all my future well-being. Many philosophers think that LCA (and other comparative accounts) is a good theory. It tracks our intuitions about death in a good way. However, as any philosophical theory, it has its problems.^14^ I will not discuss these Epicurean criticisms of Deprivationism and LCA here, but rather move on to another Deprivationist view, distinct from LCA: Gradualism.

## The badness of death: gradualism

It is important to remember that since LCA tracks the badness of death to loss of future well-being, it holds that it is, all else being equal, usually worse to die at an earlier time as opposed to a later time. If one wants to deny LCA (or Deprivationism altogether) and subscribe to the Epicurean, this feature is not much of a worry. But, we might still want to say that death is bad while pointing out some problems with this other aspect of the theory. Maybe we want to claim that the badness of death is not a strict function of the amount of well-being lost from death, but that it varies over the course of a life. Gradualist theories state exactly this. One widely discussed such theory is McMahan’s *Time-Relative Interest Account* (TRIA) (McMahan 2002, 2019). Again, on LCA, the extent of the badness of death is a function of the amount of life lost. On TRIA, the badness of death can be formulated as follows:

TRIA: The badness of death *b* at *t* is a function *b* = *(f)w* × *r* where (1) *w* is the amount of well-being lost from death and (2) *r* is the strength of the relevant relations^15^ that would have held between the individual at *t* and the same individual after *t* if the death had not occurred (McMahan 2019).

Consider these illustrating examples:

*Choice between deaths.* A day-old infant will die unless the doctor saves him. Although the infant can be saved, the condition that threatens his life cannot be cured and will certainly cause his death later around the age of thirty-five (McMahan 2002, p. 184).

To find a plausible answer here, we can simply use LCA. According to LCA, it is worse for the child to die now than to die at 35. If she dies now, she is deprived of 35 years of life 35 years is better than 0 years. McMahan then introduces a second example:

*Choice between lives.* A thirty-five-year-old woman is due to give birth the next day but there are complications with the pregnancy. The woman can live another 35 years while the fetus will die at age 35 (due to an incurable congenital condition) (McMahan 2002, pp. 185-6).

In this case, LCA is indifferent. If we save the woman, the child will lose 35 years, and the woman will gain 35 years. If we save the child, the woman will lose 35 years, and the child will gain 35 years. But McMahan thinks that *we* are not indifferent to this choice. We think that the doctor should save the woman. He further thinks that this does not depend solely on external factors, but that it is an inherent fact about the specific death: it is strictly worse for the woman to die than for the child. But LCA states that death would be equally bad for these two people. To make the point even clearer, consider:

*Generalized choice between lives*. A 15-year-old is about to die in the same hospital a newborn is about to die. Due to resource scarcity, the doctor can only save one of them. Should the doctor save the 15-year-old or the newborn?

LCA states that the doctor should save the newborn, since we can expect her loss from death to be strictly bigger (80 years compared to 65 years). But, McMahan holds, many of us think that the doctor should save the 15-year-old. The essence of this idea is that the badness of death does not bear a strict relation to the size of the loss of life.

Let’s say that we agree with McMahan. The doctor ought to save the 15-year-old because her death would be worse for her. Why is this so? McMahan says that this is because the 15-year-old has a stronger *time-relative interest* in her future. Simply put, she has a stronger connection to her future self than the newborn. The newborn has no self-consciousness or life plans, and she does not have especially strong metaphysical connections to her future self. The 15-year-old has self-consciousness, life plans, and strong metaphysical connections to her future self. Her death is bad for her, then, because it is a stronger violation of her time-relative interests. These time-relative interests in turn constitute the factor *r* in our formulation of TRIA.

TRIA has, of course, been subject to criticism, partly because it is not as widely discussed and elaborated on as LCA, and partly because some authors argue that McMahan is misguided (Broome 2019; Campbell 2019). I will set these criticisms aside, and move on to discussing TRIA and LCA in light of Absolute and Proportional Shortfall.

## Discussion

In the previous sections, I have outlined two popular theories regarding severity and two popular theories regarding the badness of death. I will now examine their relations to one another.

### The similarity between LCA and absolute shortfall

First, there seem to be clear similarities between LCA and Absolute Shortfall. To illustrate this, let us take another, closer look at Deadly disease.

*The Absolute Shortfall of deadly disease. A* condition affects 40-year-olds and has a prognosis of 5 QALYs. This patient group will then lose (80-40-5) = 35 QALYs. Another condition affects 70-year-olds, also with a prognosis of 5 QALYs. This group will lose (80-70-5) = 5 QALYs.

If we use LCA as a lens to look at this example, we get a clear verdict: since the loss for the 40-year-olds is bigger than the loss for the 70-year-olds, and the badness of death is a function of the amount of life lost because of death, death is worse for the 40-year-olds than for the 70-year-olds. It seems that LCA and Absolute Shortfall state a similar verdict. The severity of illness according to Absolute Shortfall is analogous to the *badness* of death according to LCA. The more you lose, the worse. This is the simple statement of LCA, and on further examination, it seems to be built into Absolute Shortfall. The severity that death adds to an illness is the absolute amount of QALYs lost—and, importantly—*nothing* else. Also, even if one has doubts about LCA, all Deprivationist theories bear some similarity to Absolute Shortfall, since they agree that the amount of life lost is at least *part* of the function that states the badness of death. The theories say something similar—it is worse to die the more life one loses from death. Even if LCA has its critics, it is a popular theory with good philosophical support. I would argue that this gives support to Absolute Shortfall as a theory of severity.

But, even if one wants to reject LCA and stick with TRIA, it still seems that Absolute Shortfall is the closest matching idea of severity. On both TRIA and LCA, the amount of life lost from death is at least part of what makes death bad. If death is bad in the way that LCA or TRIA claims, Absolute Shortfall is the closest theory about severity.

### The lack of support for proportional shortfall

Further, *none* of the Deprivationist accounts discussed in this article seem to give any support to the idea of Proportional Shortfall. As mentioned above, Proportional Shortfall is used as a priority setting criterion in the Netherlands, and the main argument for this is that it has “strong (political) support” (Van de Wetering et al. 2013, p. 113). It is unclear what the underlying motivations are, and it has been said that it seems “hard to defend that avoiding a full loss of all remaining health would be equally important when the choice concerns either a very large or small *absolute* QALY loss” (Van de Wetering et al. 2013). It would seem that one underlying motivation for Proportional Shortfall is that it tries to temper a supposed *ageism* that follows from Absolute Shortfall (Magnussen et al. 2015). It seems that adherents of Proportional Shortfall *would want* to say something like this: it can be equally bad (or even worse) for someone to die later in life. It all depends on the proportional size of the loss. But if we approach this idea from the viewpoint of Deprivationist theories, it seems misguided. Consider, again:

It is equally severe that a 40-year-old will die at 50 as that a 70-year-old will die at 72,5.

This is presented as an interpersonal case, involving different people, but imagine instead that it is an *intra*personal case, involving one and the same person (me). If I am 40 and then die at 50, that is of course bad for me. But, in the nearest possible world where I do not die at 50, I live to be 72,5. At 70, this death is equally bad for me as my death at 50 would have been to me at 40.^16^ This is unintuitive. Of course, my death at 72,5 is bad for me. But, all else being equal (and they are, since the currency we are dealing with is QALYs), my death at 50 *has* to be *worse*. If life after 50 has at least some well-being in it, it cannot be equally bad for me to die earlier. If death’s badness is related to severity, it is not captured by the idea of Proportional Shortfall.

Also, another underlying idea with Proportional Shortfall seems to be that it can be worse (or equally bad) to die *later*. Again, what matters is the *proportion* of life that is lost. This is strictly opposite to LCA, since LCA states that an earlier death is always a worse one (all else being equal). It also does not fit well with TRIA. On most formulations of TRIA, the Time- Relative Interest is strongest in childhood or early adolescence (for our purposes, let’s say that it is strongest at 15 years of age). From conception to this point, the time-relative interest gets stronger. After this point, it gets weaker. All else being equal, the worst time to die is at 15 (or around that age). To defend Proportional Shortfall using TRIA, one would need to formulate a version of TRIA where the time-relative interest can get stronger and stronger.

Some people believe that the same amount of life years is *worth* more to older people than to younger people. It is, they say, *very* valuable to add 2.5 years to the life of a 70-year-old. Maybe they would claim that this is *equally as good* as adding 10 years to the life of a 40-year-old.

One way to illustrate this is this: Simon uses battery-powered wireless headphones to enjoy music.^17^ Suddenly, he gets an alert on his device informing him that the battery is about to run out, and he has about 10 min of music listening left before the battery does so. It might then be reasonable to say that these 10 min are *very* important to him. He savors them and is very intentive about how he spends them. He chooses the few remaining songs carefully. It might then seem reasonable to say that these minutes are more *valuable* to him than an hour would be. Although there is something attractive about this intuition, it seems, as mentioned above, hard to find theoretical support for it. We could argue that it is false. Say for instance that after 10 min have passed, the battery of Simon’s headphones is magically refilled to its max. If those minutes were previously more valuable, they are now suddenly less valuable. This seems wrong. He now has much more time to enjoy music. How can that be a bad thing? Maybe it is bad because now he does not savor the music in the same way. But it still seems better to have one hour of battery left than 10 min. I think that the intuition that life is worth more if you have less left is one that we ought to do away with if we do not want to deal with the other, more problematic (as argued above), aspects of using Proportional Shortfall to operationalize severity.

### The lack of support for TRIA

Third, attractive as it may seem (to some), since no operationalizations of severity seem to be in line with TRIA; this gives reason to think that it would be difficult to operationalize TRIA in health care. We might think of TRIA as a good way to understand the badness of death, and intuitively, it makes some sense. But both Absolute and Proportional Shortfall go against TRIA. To see this, consider the following:

*Baby or child.* The doctor has to choose between treating a baby and treating a child. If treated, they would both live to become 80. If untreated, the baby would die at age 1, and the child would die at age 15.

TRIA would say that since death would be worse for the child, the doctor ought to treat the child. LCA would say that since death would be worse for the baby, the doctor ought to treat the baby. So far so good. With regards to severity, Absolute Shortfall says that since the QALY loss for the baby is bigger, the baby’s condition is more severe. Proportional Shortfall says that since the proportional QALY loss for the baby is bigger, the baby’s condition is more severe. Since the severity principle says that we should give priority to the patient whose condition is more severe, both Absolute and Proportional Shortfall state that the doctor ought to treat the baby. The severity principle seems to be very much in line with LCA, and not with TRIA.

Of course, this is not to say that one ought to abandon TRIA, or that it is useless in health care priority setting. TRIA is in many ways an attractive theory, especially in cases such as *Baby or child*. However, if one wants to use TRIA in health care priority setting, the work has to be done. Proponents of TRIA have to formulate a matching severity principle since there currently is no such principle available.

### The relevance of the badness of death for priority setting

Lastly, I want to discuss an argument made by Greaves (2019) that the concept of the badness of death is ill-suited for discussions of healthcare priority setting.

Greaves argues for this conclusion based on the premise that TRIA is not an axiological principle. I have my doubts about this. Greaves argues that TRIA is not a theory about badness, but that it is instead something akin to a theory about when *w*e think it is worst (as in “most upsetting”) for someone to die. We think it is more upsetting when a 15-year-old dies than when a baby dies. But McMahan clearly states that this is not the case. In his original proposal, this is not clear, but it has been clarified since. (36) Albeit in a more complicated way than LCA, TRIA tracks the badness of death. Formulating an axiological principle from TRIA might be *complicated*, but it is not impossible. Setting this aside, as I have shown there are other reasons not to use TRIA in health care priority setting, while there are good reasons to use, for instance, LCA. A good reason to use LCA is that it can give intuitive support to certain operationalizations of severity (i.e., Absolute Shortfall), and vice versa. Also, LCA can surely be formulated as an axiological principle, since LCA *clearly* tracks the badness of death. Theorizing by way of the badness of death clearly can give us a better understanding of severity and therefore be useful in health care priority setting.

Let us take stock. Deprivationist theories such as LCA provide us with a reasonable way of understanding what if bad about death (and by extension, by dying earlier). The fact that death deprives us of future well-being is what makes this the case. I have argued that if we accept this, this is a reason for us to adopt the Absolute Shortfall operationalization of severity. On this understanding, a condition is more severe the bigger the absolute loss of QALYs it causes. One implication of this is that conditions that lead to an earlier death will be (all else equal) considered more severe. Severity as a priority setting criterion urges us to give higher priority to conditions with higher severity, and hence, based on what has been argued in this article, conditions that lead to an earlier death are examples of such conditions. In our health care system, there is therefore a normative reason for more of scarce resources to be spent on curing and preventing these types of conditions. This provides support for the publicly funded health care systems that has adopted Absolute Shortfall as their operationalization of severity (such as Norway), reason for those using Proportional Shortfall (such as the Netherlands) to reevaluate, and reason for those that have not yet explicitly adopted an operationalization to do so—that of Absolute Shortfall.

## Conclusion

The connection between severity and health care priority setting on the one hand and philosophical theories about the badness of death on the other requires further attention. An attempt at this has been done in this paper. I set out with the aim of examining the possible connections between these fields. Following descriptions of two popular methods of operationalizing severity in health care priority setting, Absolute and Proportional Shortfall, as well as descriptions of two contemporary accounts of the badness of death, the Life-Comparative Account and the Time-Relative Interest Account, I have argued that the Life-Comparative Account provides the most promising underlying rationale for Absolute Shortfall. I have also argued that it is hard to find support for Proportional Shortfall by way of theories about the badness of death, and that the Time-Relative Interest, promising a theory as it is, needs further work to be suitable for use in health care priority setting. Based on this, I have also argued that the entire concept of the badness of death can be well-suited for discussions of this kind, partly because it can provide us with underlying rationales for operationalizing severity. A main takeaway from this is that Absolute Shortfall is, at least with regards to the badness of death, a more promising contender for operationalizing and thinking about severity.

## Footnotes

1 *Priority setting* should be broadly understood as all decisions regarding resource allocation in health care.

2 Henceforth *severity* (for simplicity).

3 Absolute Shortfall is used in health care priority setting in Norway (Magnussen et al. 2015). Proportional Shortfall is used in the Netherlands (van de Wetering et al. 2013). In the UK, both operationalizations are used (National Institute for Heath and Care Excellence 2022). There are other proposed ways to operationalize severity, for instance, *Prognosis* and *Lifetime Shortfall*. These are not used in practice and, arguably, not well suited for use in health care priority setting. For more, see Magnussen et al. (2015).

4 See Solberg and Gamlund (2016) for further discussion regarding the omission of Epicureanism.

5 There has been some recent discussion regarding Epicureanism’s eventual suitability in health care priority setting, see for instance Hol and Solberg (2023).

6 It is subject to debate whether dead people occupy a well-being level or not. I believe that they do not, but in this article, I take no stand on the matter. What is important for our purposes is that 1 year of non-existence equals 0 QALYs. For more on this, see Feit (2016) and Carlson and Johansson (2017).

7 The examples in this section, as well as the following, are adapted from the Norwegian report on severity and priority setting (Magnussen et al. 2015).

8 It should be noted that since the number of total expected future QALYs is not always 80, but will vary with age, this will slightly alter the exact proportion of the Proportional Shortfall. (For instance, expected future QALYs at birth might be 80, but expected future QALYs at 75 might be a higher number, say 85, since some people in the age group will have died).

9 In addition to Nagel (1970), see Bradley (2009), Broome (2004), Ekendahl (2019), Feldman (1991, 1992), Fischer (2020), Johansson (2005), Kagan (2012), Quinn (1984).

10 This is what is assumed by the termination thesis.

11 My family and friends would (hopefully) be upset by my death. My death would have a negative impact on *their* well-being.

12 For instance, I could die painlessly in my sleep. I go to bed one night and simply die in my sleep. I do not have any experience of pain. I simply do not wake up. But it could also be the case that I could die by being slowly run over by a steamroller. This will be extremely painful. Since this is an experience that I certainly *have*, the Epicurean allows it to be bad for me. And the Deprivationist agrees.

13 However, many writers have argued that Deprivationism has *implications* in these discussions.

14 The main criticisms posed against Deprivationism and LCA are The Timing Problem and The Asymmetry Problem. For discussion on these, see Johansson (2013a, 2013b), Kaufman (1995), Lucretius (1940), Nagel (1970).

15 These *relevant relations* are what McMahan calls *the time-relative interests*. What they consist of is debated, but they depend on some metaphysical relationship between the person at *t* and the same person after *t*.

16 It should be noted that this way of reasoning may suppose that we accept some view akin to *priorism*, that is, that the badness of death is chronologically *located* before death.

17 Thanks to Erik Gustavsson for this example.

## References

[CR1] Barra Matthias (2020). Severity as a priority setting criterion: Setting a challenging research agenda. Health Care Analysis.

[CR2] Belshaw Christopher (2005). 10 good questions about life and death.

[CR3] Bognar Greg (2008). Well-being and Health. Health Care Analysis.

[CR4] Bradley Ben (2009). Well-being and death.

[CR5] Bradley Ben, Johansson Jens, Feldman Fred (2013). The Oxford handbook of philosophy of death.

[CR6] Brock Dan, Murray, Christopher (2002). The separability of health and well-being. Summary measures of population health: Concepts, ethics, measurement and applications.

[CR7] Broome John (1993). Qalys. Journal of Public Economics.

[CR8] Broome John (2004). Weighing lives.

[CR9] Broome John, Gamlund, Espen, Solberg, Tollef (2019). The badness of dying early. Saving people from the harm of death.

[CR10] Broome John, Murray, Christopher (2002). Measuring the burden of disease by aggregating well-being. Summary measures of population health: Concepts, ethics, measurement and applications.

[CR11] Campbell Tim, Gamlund Espen, Solberg Carl Tollef (2019). Health care rationing and the badness of death: Should newborns count for less?. Saving people from the harm of death.

[CR12] Carlson Erik, Johansson Jens (2017). Well-being without being? A reply to Feit. Utilitas.

[CR13] DeGrazia David (2003). Identity, killing, and the boundaries of our existence. Philosophy & Public Affairs.

[CR14] DeGrazia David (2007). The harm of death, time-relative interests, and abortion. Philosophical Forum.

[CR15] Ekendahl, Karl. 2019. *The good, the bad, and the dead: An essay on well-being and death.* Doctoral Dissertation. Uppsala: Department of Philosophy, Uppsala University.

[CR16] Epicurus. , Bailey Cyril (1940). Letter to Menoceus. Oates, Whitney Jennings, trans. The Stoic and Epicurean Philosophers.

[CR17] Feit Neil (2016). Comparative harm, creation, and death. Utilitas.

[CR18] Feldman Fred (1991). Some puzzles about the evil of death. The Philosophical Review.

[CR19] Feldman Fred (1992). Confrontations with the reaper.

[CR20] Feldman Fred (2000). The termination thesis. Midwest Studies in Philosophy.

[CR21] Fischer John Martin (2020). Death, immortality, and meaning in life.

[CR22] Gamlund Espen, Solberg Carl Tollef (2014). Når er det verst å miste sitt liv? Relevansen av filosofiske grunnlagsteorier for helseprioriteringer. Norsk Filosofisk Tidsskrift.

[CR23] Gamlund Espen, Solberg Carl Tollef, Gamlund Espen, Solberg Carl Tollef (2019). Introduction: Perspectives on evaluating deaths and their relevance to health policy. Saving people from the harm of death.

[CR24] Greaves Hillary, Gamlund Espen, Solberg Carl Tollef (2019). Against “the badness of death”. Saving people from the harm of death.

[CR25] Hol Bjørn, Solberg Carl Tollef (2023). Epicurean priority-setting during the COVID-19 pandemic and beyond. De Ethica.

[CR26] Johansson Jens, Bradley Ben, Johansson Jens, Feldman Fred (2013). The timing problem. The Oxford handbook of philosophy of death.

[CR27] Johansson Jens (2013). Past and future non-existence. The Journal of Ethics.

[CR28] Johansson, Jens. 2005. *Mortal beings: On the metaphysics and value of death*. Doctoral Dissertation. Stockholm: Acta Universitatis Stockholmiensis.

[CR30] Kagan Shelly (2012). Death.

[CR31] Kaufman Frederik (1995). An answer to Lucretius’ symmetry argument against the fear of death. The Journal of Value Inquiry.

[CR32] Lucretius. , Munro Hugh Andrew, Johnstone,  (1940). On the nature of things. Oates, Whitney Jennings, trans. The stoic and epicurean philosophers.

[CR29] Magnussen Jon (2015). På ramme alvor: Alvorlighet og prioritering.

[CR33] McMahan Jeff (2002). The ethics of killing: Problems at the margins of life.

[CR34] McMahan Jeff, Gamlund Espen, Solberg Carl Tollef (2019). Saving people from the harm of death.

[CR35] Nagel Thomas (1970). Death. Noûs.

[CR36] National Institute for Health and Care Excellence. 2002. *NICE Publishes new combined methods and processes manual and topic selection manual for its health technology evaluation programmes* [NewsArticle]. NICE; NICE. https://www.nice.org.uk/news/article/nice-publishes-new-combined-methods-and-processes-manual-and-topic-selection-manual-for-its-health-technology-evaluation-programmes.

[CR37] Norheim Ole Frithjof, Gamlund Espen, Solberg Carl Tollef (2019). The badness of death: Implications for summary measures and fair priority setting in health care. Saving people from the harm of death.

[CR38] Quinn Warren (1984). Abortion: Identity and loss. Philosophy and Public Affairs.

[CR39] Solberg Carl Tollef, Gamlund Espen (2016). The badness of death and priorities in health. BMC Medical Ethics.

[CR40] Van de Wetering EJ (2013). Balancing equity and efficiency in the Dutch basic benefits package using the principle of proportional shortfall. The European Journal of Health Economics.

